# Effects of Yoga on Mental and Physical Health: A Short Summary of Reviews

**DOI:** 10.1155/2012/165410

**Published:** 2012-09-13

**Authors:** Arndt Büssing, Andreas Michalsen, Sat Bir S. Khalsa, Shirley Telles, Karen J. Sherman

**Affiliations:** ^1^Department Quality of Life, Spirituality and Coping, Center of Integrative Medicine, Faculty of Health, Witten/Herdecke University, 58313 Herdecke, Germany; ^2^Department of Internal and Complementary Medicine, Immanuel Hospital Berlin and Institute of Social Medicine, Epidemiology & Health Economics, Charité-University Medical Center, 14109 Berlin, Germany; ^3^Division of Sleep Medicine, Brigham and Women's Hospital, Harvard Medical School, Boston, MA 02115, USA; ^4^Director of Research, Patanjali Research Foundation, Haridwar 249405, India; ^5^Group Health Research Institute, Group Health Cooperative, Seattle, WA 98101, USA

## Abstract

This report summarizes the current evidence on the effects of yoga interventions on various components of mental and physical health, by focussing on the evidence described in review articles. Collectively, these reviews suggest a number of areas where yoga may well be beneficial, but more research is required for virtually all of them to firmly establish such benefits. The heterogeneity among interventions and conditions studied has hampered the use of meta-analysis as an appropriate tool for summarizing the current literature. Nevertheless, there are some meta-analyses which indicate beneficial effects of yoga interventions, and there are several randomized clinical trials (RCT's) of relatively high quality indicating beneficial effects of yoga for pain-associated disability and mental health. Yoga may well be effective as a supportive adjunct to mitigate some medical conditions, but not yet a proven stand-alone, curative treatment. Larger-scale and more rigorous research with higher methodological quality and adequate control interventions is highly encouraged because yoga may have potential to be implemented as a beneficial supportive/adjunct treatment that is relatively cost-effective, may be practiced at least in part as a self-care behavioral treatment, provides a life-long behavioural skill, enhances self-efficacy and self-confidence and is often associated with additional positive side effects.

## 1. Introduction

The conceptual background of yoga has its origins in ancient Indian philosophy. There are numerous modern schools or types of yoga (i.e., Iyengar, Viniyoga, Sivananda, etc.), each having its own distinct emphasis regarding the relative content of physical postures and exercises (*asanas*), breathing techniques (*pranayama*), deep relaxation, and meditation practices that cultivate awareness and ultimately more profound states of consciousness. The application of yoga as a therapeutic intervention, which began early in the twentieth century, takes advantage of the various psychophysiological benefits of the component practices. The physical exercises (*asanas*) may increase patient's physical flexibility, coordination, and strength, while the breathing practices and meditation may calm and focus the mind to develop greater awareness and diminish anxiety [1], and thus result in higher quality of life. Other beneficial effects might involve a reduction of distress, blood pressure, and improvements in resilience, mood, and metabolic regulation [2].

Khalsa stated that a majority of the research on yoga as a therapeutic intervention was conducted in India and a significant fraction of these were published in Indian journals, some of which are difficult to acquire for Western clinicians and researchers [3]. In their bibliometric analysis from 2004, they found that 48% of the enrolled studies were uncontrolled, while 40% were randomized clinical trials (RCT), and 12% non-RCT (N-RCT). Main categories which were addressed were psychiatric, cardiovascular, and respiratory disorders [3].

Despite a growing body of clinical research studies and some systematic reviews on the therapeutic effects of yoga, there is still a lack of solid evidence regarding its clinical relevance for many symptoms and medical conditions. For many specific indications and conditions, there is inconsistent evidence with several studies reporting positive effects of the yoga interventions, but other studies are less conclusive. In some instances, these discrepancies may result from differences between the study populations (e.g., age, gender, and health status), the details of the yoga interventions, and follow-up rates.

In this paper, we summarize the current evidence on the clinical effects of yoga interventions on various components of mental and physical health. In general, the respective reviews (Table 1) and an Agency for Healthcare Research and Quality Report (AHRQ) evidence report on “Meditation Practices for Health,” which cites also studies on yoga [30], include a heterogeneous set of studies with varying effect sizes, heterogeneous diagnoses and outcome variables, often limited methodological quality, small sample sizes, varying control interventions, different yoga styles, and strongly divergent duration of interventions. 

## 2. Yoga and Mental Health

### 2.1. Depression

We found four relevant publications, including two reviews on the effects of yoga on depression [4, 5], a description of studies on yogic breathing [6] for depression, and one “summary” [8]. The reviewing authors have reported that the studies reviewed showed a large variety of diagnoses ranging from “major depression or some other type of diagnosed depression” to “elevated depressive symptoms” [5]. Although several randomized controlled trials (RCTs) reported beneficial effects of yoga interventions for treating depressive symptoms, the quality and quantity of the data from these studies appear insufficient to conclude whether there is substantial clinical justification to consider yoga as a treatment of depression. Compared to passive controls, the yoga interventions seem to be effective; when compared with active controls, not surprisingly, the effects are less conclusive [5]. The study results are so far not sufficient in quantity and quality to determine whether studies with a focus on the *asanas* are more effective as compared to studies with meditation-focussed or *pranayama*-focussed styles. Thus, there is a strong need to conduct more conclusive studies with high methodological quality and larger patient samples. Whether motivation of depressed patients could be a problem or not remains to be clarified. There has been an attempt to explore mechanisms of action and to understand the complete picture of the effects of yoga in depression looking at electrophysiological markers of attention, and neurotransmitters which were found to change with yoga [7, 31].

### 2.2. Fatigue

We found one systematic review/meta-analysis evaluating the effects of yoga on fatigue in a variety of medical conditions. The review included 19 RCTs and included healthy persons as well as patients with cancer, multiple sclerosis, dialysis, chronic pancreatitis, fibromyalgia, and asthma [9]. Overall, a small positive effect with an SMD of 0.28 [0.24–0.33] was found. This standardized mean difference (SMD) describes the difference in the group mean values divided by the respective standard deviation; a value between 0.3 and 0.5 can be regarded as small, SMD between 0.5 and 0.8 as moderate, and SMD >0.8 as large. For those studies that included cancer patients (*n* = 10), the treatment effect of yoga was 0.20 (0.15–0.24); for all other studies that did not include cancer patients (*n* = 9), the effect was 0.46 (0.24–0.67) [9]. Nevertheless, there are some studies on cancer-related fatigue which indicate that treatment effects of yoga could be improved in well-designed future studies.

### 2.3. Anxiety and Anxiety Disorders

There is one systematic review examining the effects of yoga on anxiety and anxiety disorders [1], a Cochrane review on meditation therapy for anxiety disorders [10] (citing one yoga study [32]), a description of studies on yogic breathing (which are also addressed in the systematic review) [6], and one summary [8]. 

Most studies described beneficial effects in favour of the yoga interventions, particularly when compared with passive controls (i.e., examination anxiety), but also compared with active controls such as relaxation response or compared to standard drugs. However, there are currently no meta-analyses available which would clearly differentiate this important issue. At least the AHRQ report stated that “yoga was no better than Mindfulness-based Stress Reduction at reducing anxiety in patients with cardiovascular diseases” [30].

### 2.4. Stress

One systematic review describes the effects of yoga on stress-associated symptoms. Chong et al. identified 8 controlled trials, 4 of which were randomized, which fulfilled their selection criteria [11]. Most studies described beneficial effects of yoga interventions. Although not all studies used adequate and/or consistent instruments to measure stress, they nevertheless indicate that yoga may reduce perceived stress as effective as other active control interventions such as relaxation, cognitive behavioural therapy, or dance. 

Also the AHRQ report stated that “yoga helped reduce stress” [30]. Here, the two included studies showed a significant reduction of stress scores in favour of the yoga group (SMD = −1.10 [CI: −1.61 to −0.58].

**Posttraumatic Stress Disorder**

A single review article looked at the existing research on yoga for posttraumatic stress disorder (PTSD) [12]. Seven articles were reviewed which included 8 studies on PTSD following exposure to natural disasters such as a tsunami and a hurricane (1 RCT, 1 N-RCT, 3 group study, 2 single-arm studies, 1 cross-sectional study) and 2 studies on PTSD due to combat and terrorism (1 RCT, 1 single-arm study). After a natural disaster, yoga practice was reported to significantly reduce symptoms of PTSD, self-rated symptoms of stress (fear, anxiety, disturbed sleep, and sadness) and respiration rate. Similarly, yoga interventions were able to improve the symptoms of PTSD in persons with PTSD after exposure to combat and terrorism. The interventions varied in duration from one week (when interventions were given on the site) to six months. The review suggested a possible role of yoga in managing PTSD, though long-term studies conducted with greater rigor are needed [12].

## 3. Yoga and Physical Fitness

### 3.1. Physical Fitness

There was one critical review which evaluated whether yoga can engender fitness in older adults [13]. Ten studies with 544 participants (mean age 69.9 ± 6.3) were included; 5 of these studies were RCTs, and 5 studies had a single-arm pre/post-design. With respect to physical fitness and function, the studies reported moderate effect sizes for gait, balance, body flexibility, body strength, and weight loss [13]. However, there is still a need for additional research trials with adequate control interventions (active and specific) to verify these promising findings. 

One may expect that retaining physical fitness and improving physical functioning can have a positive effect on functional abilities and self-autonomy in older adults. Further studies should address whether or not individuals' self-esteem and self-confidence will increase during the courses, and whether or not regular classes may also improve social competence and involvement. A problem with studies enrolling elderly subjects can be compliance with the study protocol leading to low levels of study completion and long-term follow-up data. Future studies should investigate the most appropriate duration of yoga intervention and the most suitable postures and yoga style for the elderly. 

### 3.2. Sympathetic/Parasympathetic Activation

There were 42 studies on the yoga effects on sympathetic/parasympathetic activation and cardiovagal function [14], that is, 9 RCTs, 16 non-RCTs, 15 uncontrolled trials, and 2 cross-sectional trials. Most studies offered “some evidence that yoga promotes a reduction in sympathetic activation, enhancement of cardiovagal function, and a shift in autonomic nervous system balance from primarily sympathetic to parasympathetic” [14]. However, some of the studies included in the review showed less clear-cut or even contrasting, effects. Because most of these effects are short-term phenomena, more rigorous work is needed. 

Another lacuna is that there are very few studies which have studied plasma catecholamine levels and most of them are early studies [33, 34].

### 3.3. Cardiovascular Endurance

Raub's literature review, which included 7 controlled studies, reported “significant improvements in overall cardiovascular endurance of young subjects who were given varying periods of yoga training (months to years)” [15]. Outcome measures included oxygen consumption, work output, anaerobic threshold, and blood lactate during exercise testing. As expected, physical fitness increased in adolescents or young adults (athletes and untrained individuals) compared to other forms of exercise, with a longer duration of yoga practice resulted in better cardiopulmonary endurance. 

## 4. Yoga and Cardiopulmonary Conditions

### 4.1. Blood Pressure and Hypertension

Innes et al. reported on 37 studies investigating the effects of yoga on blood pressure and hypertension, among them 12 RCTs, 12 nonrandomized clinical trials, 11 uncontrolled studies, 1 cross-sectional study, and 1 single yoga session examination. Most reported a reduction of systolic and/or diastolic pressure. However, there were several noted potential biases in the studies reviewed (i.e., confounding by lifestyle or other factors) and limitations in several of the studies which makes it “difficult to detect an effect specific to yoga” [14]. 

Ospina et al.'s AHRQ cites two studies which found small, insignificant improvements of systolic (weighted mean difference = −8.10; 95% CI, −16.94 to 0.74) and diastolic blood pressure (weighted mean difference = −6.09; 95% CI, −16.83 to 4.64) in favour of yoga when compared to no treatment [30]. When compared to health education, yoga interventions resulted only in small and insignificant improvements of systolic blood pressure (weighted mean difference = −15.32; 95% CI, −38.77 to 8.14) and diastolic blood pressure (weighted mean difference = −11.35; 95% CI, −30.17 to 7.47) [30].

### 4.2. Pulmonary Function

In his descriptive literature review, Raub also examined studies evaluating yoga's effects on lung function in healthy volunteers and patients with chronic bronchitis and asthma [15]. In healthy volunteers practicing yoga, there are reported improvements of various parameters of lung function with breathing control techniques, specific postures, and/or relaxation techniques [15]. However, these improvements were “not consistent and depended upon the length of yoga training, the type of yoga practice used (e.g., breathing exercises and yoga postures), and the type of subject” [15]. Raub also cited some studies on patients with asthma describing improvements in peak expiratory flow rate, medication use and asthma attack frequency. In a double-blinded RCT with placebo-control, [35] there were only a few small and insignificant improvements in lung function variables. Thus, more rigorous trials are needed to clarify the value of yoga breathing practices for patients with asthma. 

## 5. Yoga and Metabolic/Endocrine Conditions

### 5.1. Glucose Regulation

Three systematic reviews examined the effects of yoga on risk indices associated with insulin resistance syndrome [14], risk profiles in adults with type 2 diabetes mellitus [16], and the management of type 2 diabetes mellitus [17]. Innes et al. [14] identified several studies on the effects of yoga on insulin resistance syndrome-associated variables, that is, 2 RCTs, 2 non-RCTs, and 8 uncontrolled clinical trials. These studies reported postintervention improvement in various indices in adults. However, the results varied by population (healthy adults, adults at cardiovascular disease risk, adults with type 2 diabetes, etc.) and study design. 

Another systematic review by Aljasir et al. [17] addressed the management of type 2 diabetes mellitus and concluded that the reviewed trials “suggest favourable effects of yoga on short-term parameters related to diabetes but not necessarily for the long-term outcomes.” However, the duration of treatment in the reviewed studies was variable (ranging from 20 min. session per day to three to five 90 min. sessions in the review of Aljasir et al. [17]; 3-4 h per day for 8 days, 2 sessions per day (25–35 min) for 3 months to 40 min per day for 6 months, and 72 4 h sessions during 12 months in the review by Innes and Vincent [16]).

The AHRQ cites two studies comparing yoga versus medication which reported a large and significant reduction of fasting glucose in individuals with type 2 diabetes in one study, and a smaller but still significant improvement in the other study [30]. The authors discussed differences in the study populations, and interventions as possible explanation for the observed heterogeneity of results. 

### 5.2. Menopausal Symptoms

A single review addressed menopausal symptoms and analyzed 3 RCT, 1 N-RCT, and 3 uncontrolled clinical trials [18]. Although some studies reported beneficial effects, “the evidence was insufficient to suggest that yoga is an effective intervention for menopause” [18]. 

A recent systematic review included 5 RCTs, which addressed effects of yoga on menopausal symptoms, particularly psychological symptoms, somatic symptoms, vasomotor symptoms, and/or urogenital symptoms [19]. However, yoga was associated with small effects on psychological symptoms (SMD = −0.37; 95% CI −0.67 to −0.07; *P* = 0.02), but no effects on “total menopausal symptoms, somatic symptoms, vasomotor symptoms, or urogenital symptoms” [19].

## 6. Yoga and Musculoskeletal Conditions

### 6.1. Musculoskeletal Functioning and Pain

There were 3 systematic reviews [20–22] and 2 other reviews on the effects of yoga on musculoskeletal function, chronic pain conditions, and pain-associated disability [23, 24]. Two reviews specifically addressed low back pain [22, 24] or arthritis [23], while the other reviews summarized studies on various chronic pain conditions, most with a focus on musculoskeletal conditions and associated disability. 

Posadzki et al. [21] included 11 RCTs with variable methodological quality and found that 10 of 11 studies reported significantly greater effects in favor of yoga when compared to “standard care, self care, therapeutic exercises, relaxing yoga, touch and manipulation, or no intervention.” A recent meta-analysis on pain intensity/frequency, and pain-related disability included 5 RCTs with single blinding, 7 RCTs without blinding, and 4 non-RCTs [20]. Reviewed studies included yoga for the treatment of back pain (6 studies), rheumatoid arthritis (2 studies), headache/migraine (2 studies), and other indications (i.e., hemodialysis, irritable bowel syndrome, labor pain, etc.). All of these studies reported positive effects in favor of the yoga interventions. There were moderate treatment effects with respect to 5 pain (SMD = −0.74 [CI: −0.97 to −0.52], *P* < 0.0001), and pain-related disability (SMD = −0.79 [CI: −1.02 to −0.56], *P* < 0.0001). Despite some study limitations, there was evidence that yoga may be useful for several pain-associated disorders. Thus, well-designed larger scale studies with adequate controls for confounding factors and more thorough statistical analyses are needed to verify these promising findings. 

With respect to chronic back pain, the studies indicate that yoga was more effective than the control interventions (including usual care or conventional therapeutic exercises), albeit some studies showed no between group difference [22]. Two recent and properly powered trials of yoga for back pain were published and reported clinically meaningful benefits for yoga over usual medical care from 6- to 12-months postrandomization [36, 37], but not over an intensive stretching intervention [36].

## 7. Yoga and Specific Diseases

### 7.1. Cancer

With respect to cancer, there are 2 reviews [25, 26] and 2 meta-analyses (one with 10 studies [27], and one “letter to the editor” with 6 studies [28]). According to the findings of the more comprehensive meta-analysis of Lin et al., the yoga groups showed improvements in psychological health when compared to waitlist or supportive therapy groups, that is, anxiety (8 studies: SMD = −0.76 [−1.34 to −0.19], *P* = 0.009), depression (8 studies: SMD = −0.95 [−1.55 to −0.36], *P* = 0.002), distress (2 studies: SMD = −0.4 [−0.67 to −0.14], *P* = 0.003), and stress (5 studies; SMD = −0.95 [−1.63 to −0.27], *P* < 0.006) [27]. With respect to overall quality of life, there was just a trend towards improvement (SMD = −0.29 [−0.58 to 0.001], *P* = 0.06). To explain the positive outcomes, Smith and Pukall suggested that complex pathways which may involve relaxation, coping strategies, acceptance, and self-efficacy [26]. Although Lin et al. stated that the “findings are preliminary and limited and should be confirmed through higher-quality, randomized controlled trials,” they nevertheless attested “potential benefit of yoga for people with cancer in improvements of psychological health” [27]. However, the outcome parameters described in these cancer reviews were also addressed in the symptom-specific reviews described above. 

### 7.2. Epilepsy

To assess the potential effects of yoga in the treatment of epilepsy, 1 Cochrane review analyzed 1 RCT and 1 N-RCT [29]. However, the authors were not able to draw “reliable conclusions” whether yoga may be effective or not. 

## 8. Discussion

These reviews suggest a number of areas where yoga may be beneficial, but more research is required for virtually all of them to more definitively establish benefits. However, this is not surprising given that research studies on yoga as a therapeutic intervention have been conducted only over the past 4 decades and are relatively few in number. Typically, individual studies on yoga for various conditions are small, poor-quality trials with multiple instances for bias. In addition, there is substantial heterogeneity in the populations studied, yoga interventions, duration and frequency of yoga practice, comparison groups, and outcome measures for many conditions (e.g., depression and pain). Disentangling the effects of this heterogeneity to better understand the value of yoga interventions under various circumstances is challenging. For many conditions, heterogeneity and poor quality of the original trials indicated that meta-analyses could not be appropriately conducted. Nevertheless, some RCTs of better quality found beneficial effects of yoga on mental health (see Uebelacker et al.'s critical review [5]). Further investigations in this area are recommended, particularly because of the plausibility of the underlying psychophysiological rationale (including the efficacy of frequent physical exercises, deep breathing practices, mental and physical relaxation, healthy diet, etc.). 

While it is not surprising that physical fitness can be improved by training, using either yoga or conventional exercises, it is of interest that in individuals with pain yoga may have beneficial effects with overall moderate effects sizes. However, these effects were strong particularly in healthy individuals, but much weaker in patients with chronic pain conditions. The beneficial effects might be explained by an increased physical flexibility, by calming and focusing the mind to develop greater awareness and diminish anxiety, reduction of distress, improvement of mood, and so forth. Because patients may recognize that they are able to be physically active, even despite of persisting pain symptoms, they may therefore experience higher self-competence and self-awareness, which contributes to higher quality of life.

Conceivably, *asanas* particularly have a positive effect on fitness and physical flexibility with a secondary effect on the mental state, while the *pranayama* practices and relaxation/meditation techniques may result in greater awareness, less stress, and higher well-being and quality of life. However, this remains to be shown in well-performed future studies. 

Because patients are engaged in the yoga practices as a self-care behavioural treatment, yoga interventions might well increase self-confidence and self-efficacy. On the other hand, patients with psychological burdens and/or low motivation (i.e., depression, anxiety, fatigue, etc.) might be less willing to participate fully in intensive yoga interventions. Some of these studies found relatively low participation and high dropout rates in some of the analysed studies. Patient compliance may be higher with the social support within group interventions, while private regular practices at home might be more difficult to perform consistently. These factors need to be addressed in further studies. Innes et al. [14] argued that most studies were from India where “yoga is an integral part of a longstanding cultural and spiritual tradition.” It is thus unclear whether adherence in Western patients might be the same. Many of the Indian clinical trials, which have been conducted in residential settings, not typically found outside India, include yoga class interventions 5 to 7 days per week, whereas such compliance would not be possible with patient populations outside India. However, such practices are unlikely to be continued, at least at such intensity. If as believed by some yoga practitioners, the intensity of the practice should be greater at the beginning of therapy, such programs would be an excellent way to begin yoga treatment. In India, there is a gradual shift in the attitude towards yoga with most urban Indians under the age of 35 believing yoga is a way to keep fit rather than attaching the same cultural importance to it, which earlier generations did. For these reasons, cross-cultural studies (which are lacking) using an identical intervention given to a population in India and parallel conducted elsewhere would be very useful. 

Motivation might be a crucial point. To overcome this, shorter time interventions might be an option for some specific indications (i.e., pain and depressive symptoms), while the cardiovascular and fitness effects might require long-term practices. In fact, some pain studies suggest that short-term interventions might be more effective than longer durations of practice [20]. This would indicate a putative lack of motivation to be physically active. Indeed, a couple of reviews noted that data on subject treatment compliance was not routinely reported in most studies [4, 30].

Clearly yoga intervention programs require an active participation of the individuals as do all behavioral interventions, and thus adherence might be a crucial point that limits potentially beneficial effects of yoga. It is apparent in many life style diseases, that patients must change attitudes and behaviour in order to successfully treat these diseases. A positive feature of yoga interventions is that they may in fact be very supportive for the execution and maintenance of such lifestyle changes due to the experience of well-being from the practices which can support regular practice, and from the changes in mind/body awareness that occur over time with continued yoga practice, which will in turn support a desire to adopt and maintain healthy behaviours.

Thus, further studies should identify which patients may benefit from the interventions, and which aspects of the yoga interventions (i.e., physical activity and/or meditation and subsequent life style modification) or which specific yoga styles were more effective than others. Larger-scale and more rigorous research is highly encouraged because yoga may have potential to be implemented as a safe and beneficial supportive/adjunct treatment that is relatively cost-effective, may be practiced at least in part as a self-care behavioral treatment, provides a life-long behavioural skill, enhances self-efficacy and self-confidence, and is often associated with additional positive side effects (Table 2).

The degree to which yoga interventions are curative treatments remains to be determined; currently it is safe to suggest that yoga can be a beneficial supportive add-on or adjunct treatment. Jayasinghe stated that one may “conclude that yoga can be beneficial in the primary and secondary prevention of cardiovascular disease and that it can play a primary or a complementary role in this regard” [38]. Because of yoga's low risk for side effects, when selecting appropriate postures for the population, and potential for actual positive side effects, it might be a promising candidate particularly for cardiac rehabilitation, depending on the patients' abilities and willingness to adopt yoga practices with regularity. However, the meditative and self-reflective (cognitive) aspects of yoga could be problematic especially for patients with psychotic or personality disorders. Nevertheless, there is currently insufficient data on contraindications or side effects related to yoga practices in patients with psychological disorders. 

Taken together, while several reviews suggest positive benefits of yoga, various methodological limitations (including small sample sizes, heterogeneity of controls and interventions) limit the generalizability of these promising study findings. It is quite likely that yoga may help to improve patient self-efficacy, self-competence, physical fitness, and group support, and may well be effective as a supportive adjunct to mitigate medical conditions, but not yet as a proven stand-alone, curative treatment. Confirmatory studies with higher methodological quality and adequate control interventions are needed.

## Figures and Tables

**Table 1:** Systematic reviews for the different domains discussed.

Indications	Studies on yoga interventions
Depression	2 reviews [4, 5], 1 description of studies on yogic breathing [6, 7], 1 summary [8]
Fatigue	1 systematic review [9]
Anxiety and anxiety disorders	1 systematic review [1], 1 Cochrane review on meditation therapy [10], 1 description of studies on yogic breathing [6, 7], 1 summary [8]
Stress	1 systematic review [11]
Posttraumatic stress disorder	1 review article [12]
Physical fitness	1 critical review [13]
Sympathetic/parasympathetic activation	1 systematic review [14]
Cardiovascular endurance	1 review [15]
Blood pressure and hypertension	1 systematic review [14]
Pulmonary function	1 review [15]
Glucose regulation	3 systematic reviews [14, 16, 17]
Menopausal symptoms	1 review [18], 1 systematic review [19]
Musculoskeletal functioning and pain	3 systematic reviews [20–22], 2 reviews [23, 24]
Cancer	2 reviews [25, 26], 2 meta-analyses [27, 28]
Epilepsy	1 Cochrane review [29]

**Table 2:** Level of action and observed effects of yoga interventions.

	Specific effects	Unspecific effects
Cognition	Contemplative states; Mindfulness; Self-identity; Self-efficacy; Beliefs; Expectations	Control of attentional networks
Emotions	Emotional control/regulation	Quality of Life
Physiology	Vagal afferent activity; Heart rate/Respiratory; Relaxation response/Stress reduction	Social contacts
Physical body	Physical flexibility, Fitness/Endurance	Healthy life style

Specific and unspecific effects are often interconnected.
